# Dynamical Observation on Biological Progression of VX2 Liver Tumors to Identify the Optimal Time for Intervention in Animal Models

**DOI:** 10.1371/journal.pone.0074327

**Published:** 2013-08-16

**Authors:** Zhenguang Wang, Guangjie Yang, Pei Nie, Junhua Fu, Xufu Wang, Dan Liu

**Affiliations:** 1 PET/CT Room, the Affiliated Hospital of Qingdao University Medical College, Qingdao University, Qingdao, China; 2 Department of CT, Shandong Medical Imaging Research Institute, Shandong University, Jinan, China; 3 Department of Interventional Medicine, the Affiliated Hospital of Qingdao University Medical College, Qingdao University, Qingdao, China; 4 Department of Nuclear Medicine, the Affiliated Hospital of Qingdao University Medical College, Qingdao University, Qingdao, China; 5 Department of Radiology, Laigang hospital, Laiwu, China; 6 Department of Nuclear Medicine, Qilu hospital, Shandong University, Jinan, China; Northwestern University Feinberg School of Medicine, United States of America

## Abstract

**Purpose:**

Based on practice guideline of “management of hepatocellular carcinoma (HCC): update” published by American Association for the Study of Liver Diseases (AASLD) and “Barcelona Clinic Liver Cancer staging system (BCLC),” this study investigated how to enroll the optimal VX2 liver tumor model for HCC researches by dynamically observing the biological progression of the tumor.

**Materials:**

Thirty-two healthy New Zealand white rabbits were implanted VX2 liver tumor by cell suspension method (n=24) and tissue fragment method (n=8). All the rabbits underwent CT scans on day 7, 14, 21 and 28 after implantation to observe the size of the tumors, the time when metastases and ascites occurred and the survival time. Appropriate intervention times were estimated corresponding to different clinical HCC stages by using tumor diameter-time curve.

**Results:**

The VX2 liver tumors grew rapidly within 28 days after implantation. And the tumors in the cell suspension group grew faster than those of the tissue fragment group. The appropriate intervention time corresponding to very early stage, early stage and intermediate stage were <11 days, 11–16.9 days and >16.9 days, respectively in the cell suspension group, and <19.9 days, 19.9–25.5 days and >25.5 days, respectively in the tissue fragment group.

**Conclusion:**

Preclinical animal research needs to improve on different levels to yield best predictions for human patients. Researchers should seek for an individualized proposal to select optimal VX2 liver tumor models for their experiments. This approach may lead to a more accurate determination of therapeutic outcomes.

## Introduction

The VX2 liver tumor model has been widely and efficiently used on the therapeutic response of various treatment, mainly including percutaneous ablation, transarterial embolization and chemoembolization, drug treatment and comprehensive treatment [1–5]. For human patients, the prognosis of solid tumors is generally related to tumor stage at presentation, and treatment decisions are also guided by tumor stage [6]. However, the experimental models were usually inappropriately selected in many studies using VX2 tumor models. They are enrolled approximately 2 weeks after tumor transplantation [7–10] rather than being considered the stages of their clinical counterparts. Nowadays, the increased measurement precision in preclinical research has somewhat raised expectations regarding human outcome prediction of animal data [11].

In this pilot study, based on practice guideline of “management of hepatocellular carcinoma (HCC): update” [6] published by American Association for the Study of Liver Diseases (AASLD) and “Barcelona Clinic Liver Cancer staging system (BCLC)”, we investigated how to enroll the optimal VX2 liver tumor models for HCC researches by dynamically observing the biological progression of the tumors.

## Materials and Methods

### Animals

The animal experiments were performed in accordance with the protocol approved by the animal care committee of the Affiliated Hospital of Medical College Qingdao University and were in compliance with institutional guidelines (Permit Number: 2012-0087). All surgery was performed under anesthesia with sodium pentobarbital or a mixture of ketamine hydrochloride and diazepam, and all efforts were made to minimize suffering. Thirty-two New Zealand white rabbits (supplied by Qingdao Institute of Materia Medica, Qingdao, China) weighing 2.7 kg-3.2 kg were divided into 2 groups by means of random number table. The study’s primary endpoint was overall survival.

### Preparation of VX2 cell suspension and VX2 tissue fragments

The VX2 tumor-bearing rabbit (obtained from Qingdao University, Qingdao, China) was sacrificed under anesthesia with intravenous injection of sodium pentobarbital (30 mg/kg body weight) when the tumor grew to a size of about 4 cm in diameter. Immediately after sacrifice of the tumor-bearing rabbit the tumor was harvested and put into 0.9% sodium chloride. The tumor was stripped to obtain flesh hoary fish-meat like tissue. Then the necrosis tissue, the surrounding connective tissue and fat were removed. For tissue fragments, the chunks of the excised tumor were cut into pieces of 1.0-2.0 mm^3^ fragments mechanically with ophthalmological scissors. For cell suspension, the tissue fragments were minced in 4°C physiological buffered saline and then filtered through an iron mesh with 0.08 mm^2^ pores to remove macroscopic tissue fragments. The filtrate was centrifuged at 2000 rpm for 5 min under room temperature and resuspended to a concentration of 1×10^7^ cells/ml using physiological buffered saline. Trypan blue was used to evaluate the viability of the VX2 carcinoma cells. All procedures were performed in a laminar flow cabinet using the aseptic technique.

### VX2 liver tumor implantation

Thirty-two healthy New Zealand white rabbits divided into 2 groups were implanted. In the cell suspension group (n=24), the VX2 cell suspension was injected in a volume of 0.1ml with a 25-gauge needle. In the tissue fragment group (n=8), two fragments of VX2 tumor were implanted with an 18-gauge needle.

**Figure 1:**
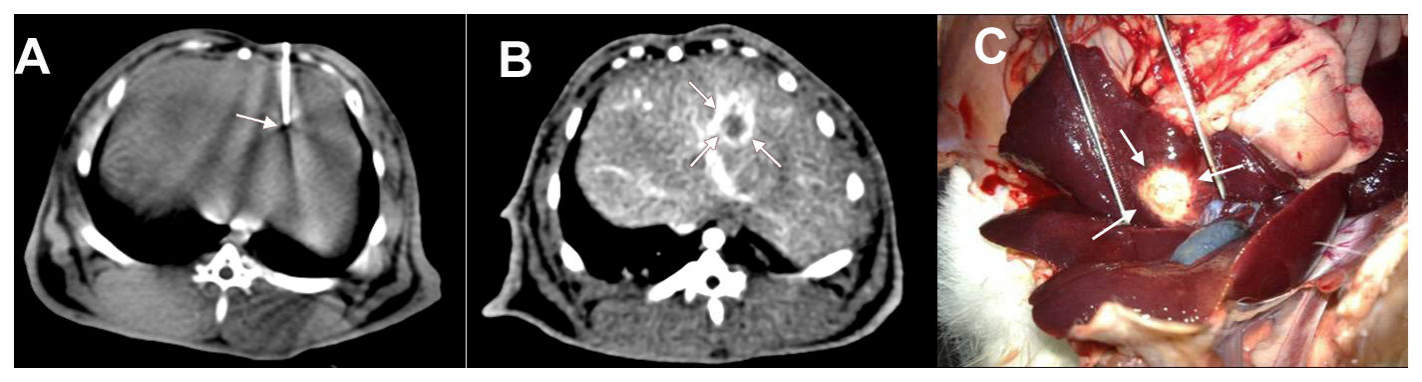
Tumor implantation, CT scans and macroscopic appearance of an individual rabbit of the cell suspension group. A shows tumor implantation under CT guidance and transplantation site (white arrow); B shows the tumor on contrast-enhanced CT scan (white arrow) on day 14; C shows a single tumor in the left medial lobe of liver during dissection on day 32 after implantation (white arrow).

Prior to all procedures, anesthesia was induced by intramuscular injection of a mixture of ketamine hydrochloride (35 mg/kg body weight) and diazepam (5 mg/kg body weight). Anesthesia was maintained with repeated intramuscular injections of diazepam when necessary. All the rabbits were implanted VX2 cell suspension or tissue fragments by percutaneous CT guided implantation method (Figure 1.A). The abdomen of each recipient rabbit was shaved and disinfected with ethanol and povidone iodine. CT scans were performed after fixing the rabbit on the domestic operative table at supine position. Individual tumor implantation plan containing site, angle, and depth of puncture was made based on the CT images. Then according to the implantation plan and experiment design, each recipient rabbit underwent CT scans again and was implanted VX2 cell suspension or tumor fragments to the left medial lobe of the liver. The puncture site was gently compressed for three minutes using an alcoholic cotton gauze to prevent bleeding and leakage after removal of the needle. The day of the tumor inoculation was considered day 0.

### CT examination

CT scans on all animals were performed with the 64-slice CT (Sensation 64, Siemens Medical System) on day 7, 14, 21 and 28. The parameters of CT scans were as follows: 100 kV tube voltage, 100 mAs tube current, 512×512 matrix, 1.25-mm section thickness. To reduce the respiratory movement, an abdominal bandage was fixed during CT examination. An intravenous infusion needle was inserted into the auricular vein for iodinated contrast medium (Schering Ultravist, Iopromide, 350 mg I/mL, Berlin, Germany) injection at a volume of 2 mL/kg body weight. Contrast-enhanced CT scans were started at 10s, 30s and 100s after initiation of contrast agent injection, and the injection rate was 1 mL/s. Anesthesia was maintained as described above during the CT examinations.

The presence of tumor was confirmed with plain and contrast-enhanced CT scans. On day 21, the rabbits with no tumor shown on CT were sacrificed and dissected for validation.

To evaluate the size of the tumors, the volume and the tumor diameter were determined from CT measurements. The tumor volume (V) was calculated according to the equation V=L×S×S/2 [12]. L was the longest diameter, which was described as the tumor diameter in our study, and S was the shortest diameter of the tumor. Two radiologists performed the measurements in consensus.

### Statistical analysis

SPSS version 17.0 software (SPSS Inc, Chicago, IL) was used to evaluate statistical differences. Results were expressed as means ± standard deviations for quantitative variables and as frequencies or percentages for categorical variables. Differences between two groups were assessed with independent Student’s t-test and Fisher exact test. Two-tailed P < 0.05 was considered statistically significant.

## Results

Tumor implantation succeeded in thirty of all thirty-two animals confirmed by CT scans. Dissection of the other two rabbits confirmed that there was no tumor in liver on day 21. Two rabbits died on day 23 and 26 before the 4th CT scans. We attributed the deaths to respiratory failure from bilateral pleural effusion and lung metastases as confirmed at autopsy. Eight successfully implanted rabbits including six (on day 14) of the cell suspension group and two (on day 21) of the tissue fragment group were selected for another experiment of our series study. Part of the analytical data was based on the remainder of the animal models. All the data used for independent Student’s t-test accorded with normal distribution (P>0.05).

### The rate of tumor visualization on CT scans

On CT scans, only 12.5% (4/32) and 28.13% (9/32) of the implanted tumors were identified by plain CT scans on day 7 and 14 (Figure 1.B). On contrast-enhanced CT scans, the rate of tumor visualization was higher. The rate of tumor visualization on plain and contrast-enhanced CT scans increased with the time after implantation (Table 1).

**Table 1:** Tumor visualization rate on CT scans after implantation.

	Plain scan (%)	Contrast-enhanced scan (%)
Day 7	12.50 (4/32)	53.13 (17/32)
Day 14	28.13 (9/32)	75 (24/32)
Day 21	57.69 (15/26)	73.08 (19/26)
Day 28	100 (20/20)	100 (20/20)

### Tumor size

**Figure 2:**
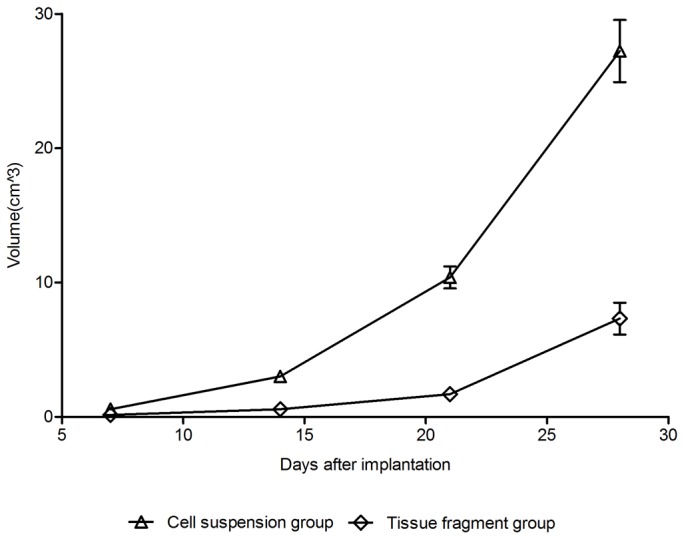
Time-tumor volume curves of the cell suspension group and the tissue fragment group after implantation.

The size of the tumors in both groups during 28 days after the implantation is summarized in Table 2. The tumors enlarged rapidly in both groups (Figure 2). Compared with the cell suspension group, the diameter and the volume were significantly smaller in the tissue fragment group (P<0.05).

**Table 2:** Comparison of the tumor diameter and volume between the cell suspension group and the tissue fragment group after implantation.

			Day 7				Day 14					Day 21					Day 28				
	Diameter (cm)		Volume (cm^3^)		Diameter (cm)			Volume (cm^3^)		Diameter (cm)			Volume (cm^3^)		Diameter (cm)			Volume (cm^3^)	
Cell suspension group		1.39±0.13		0.57±0.15		2.43±0.26			3.01±0.90		3.80±0.43			10.38±3.47		5.15±0.47			26.62±10.26	
Tissue fragment group		0.97±0.00		0.15±0.08		1.46±0.11			0.57±0.21		2.10±0.28			1.70±0.54		3.45±0.47			7.32±2.36	
*T*		4.59		3.90		8.12			5.98		9.10			6.01		6.45			3.67	
*P*		0.000		0.001		0.000			0.000		0.000			0.000		0.000			0.002	

**Figure 3:**
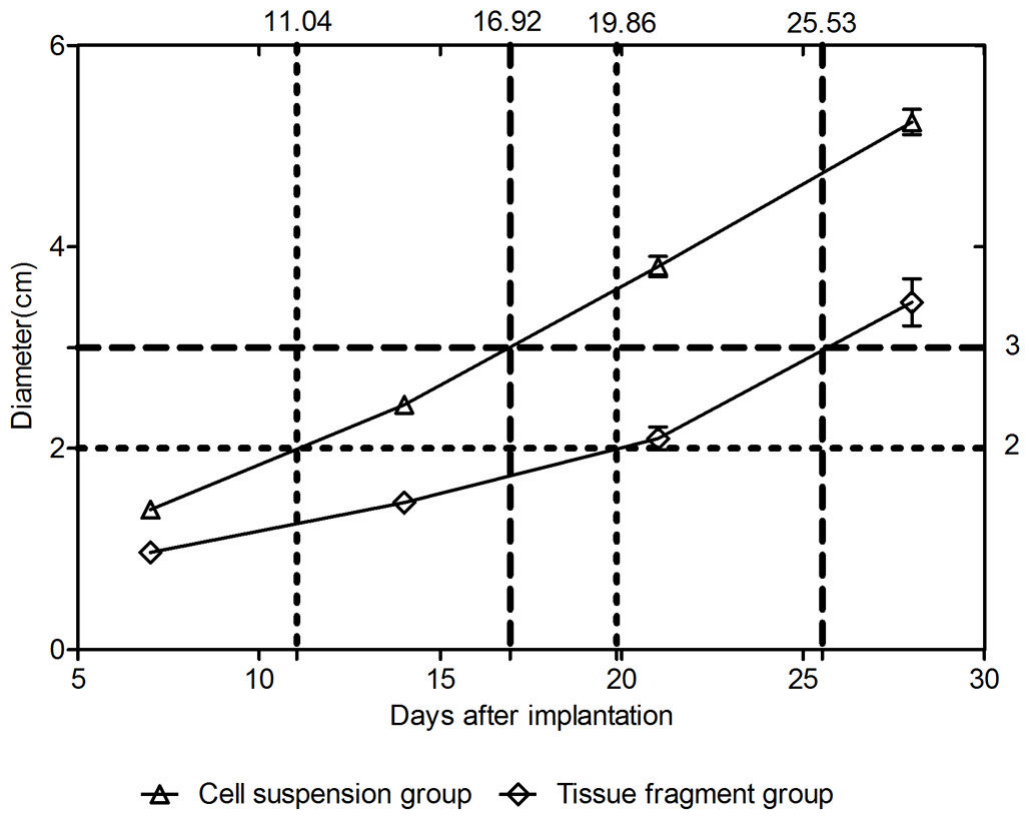
Time-tumor diameter curves of the cell suspension group and the tissue fragment group after implantation. Six reference lines were used to estimate the growth time of tumor diameter corresponding to 2 cm and 3 cm.

Based on the BCLC system, the tumor diameter of very early stage, early stage and intermediate stage is < 2 cm, 2-3 cm and > 3 cm, respectively. To identify the time after implantation corresponding to the diameter of 2 cm and 3 cm was of great importance for the initiation of intervention. The time-tumor diameter curves obtained by SPSS software were used to estimate the growth time of tumor diameter corresponding to 2 cm and 3 cm (Figure 3). The growth time of tumor diameter corresponding to 2 cm and 3 cm was 11 days and 16.9 days, respectively by cell suspension method, and 19.9 days and 25.5 days, respectively by tissue fragment method.

The observation suggested that the Performance Status (PS), the mental status and the appetite of the rabbits were normal before the tumors growing to 2 cm, and then deteriorated rapidly with the progression of the tumors when they were larger than 3 cm.

### Metastases and ascites

**Figure 4:**
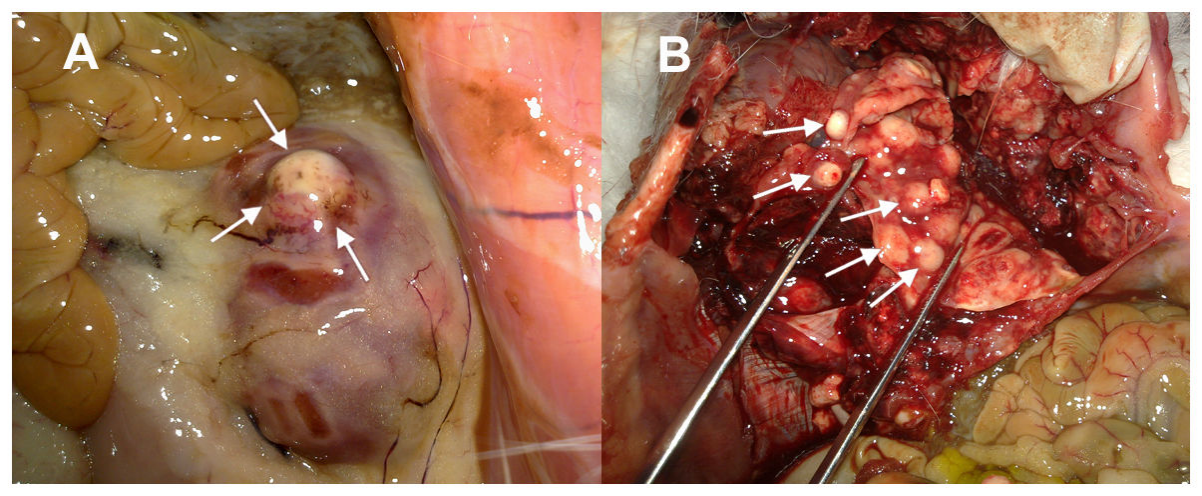
The macroscopic appearance of extra-hepatic metastases. A shows the renal metastases on day 42 after dissection of a rabbit in the cell suspension group (white arrow); B shows multiple pulmonary metastases on day 44 after dissection of another rabbit in the cell suspension group (white arrow).

**Figure 5:**
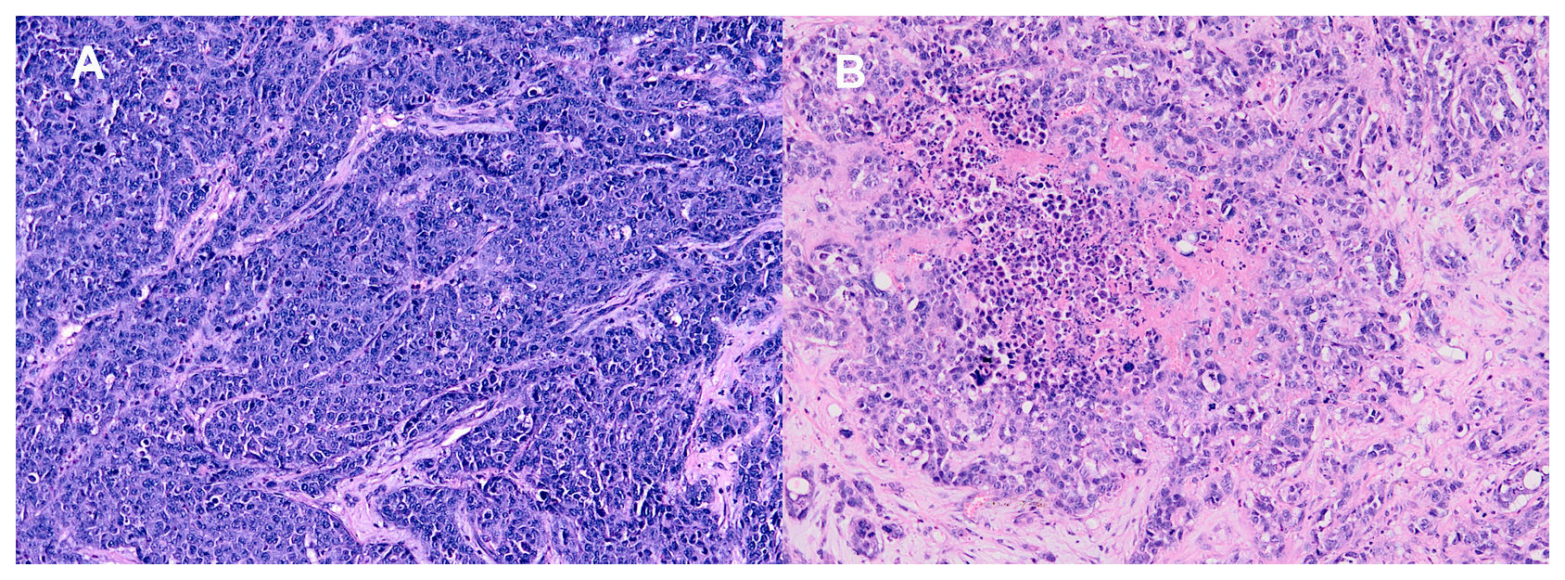
Histopathologic appearance of VX2 liver tumors (H&E, ×100). A shows Viable VX2 cells with obvious nuclear atypia in the periphery of VX2 liver tumor. B shows tumor necrosis in the center of VX2 liver tumor with large size.

Metastases (Figure 4) and ascites detected in both groups after implantation are summarized in Table 3. A very low metastases rate (1/24) was shown in the cell suspension group on day 14. Then the occurrence rate of metastases and ascites increased rapidly. Until day 28, no metastases and ascites were shown in the tissue fragment group. Moreover, necrosis was usually detected in the center of the tumors with large size (Figure 5).

**Table 3:** Metastases and ascites in the cell suspension group and the tissue fragment group after implantation.

			Day 7					Day 14					Day 21					Day 28				
	Cell suspension group			Tissue fragment group		Cell suspension group			Tissue fragment group		Cell suspension group			Tissue fragment group		Cell suspension group			Tissue fragment group		
Intra-hepatic metastases		0			0		1			0		4			0		12			1
Renal metastases		0			0		0			0		1			0		4			0
Pulmonary metastases		0			0		1			0		9			0		10			0
Ascites		0			0		0			0		2			0		3			0

### Survival time

Animals in both groups survived more than 5 weeks after implantation in average, with 35.94 ± 9.99 days for the cell suspension group, and 41.00±12.57 days for the tissue fragment group respectively. There was no statistically significant difference in survival time between the two groups (P>0.05).

## Discussion

Preclinical animal research needs to improve on different levels to yield best predictions for human patients, and animal models of diseases need to be as reliably reflective of the patient’s situation as possible [11]. Therefore, to obtain more comparable and informative data for human situation, the optimal animal models which are most similar to clinical situations should be selected [13,14]. However, how to enroll the most similar VX2 tumor models to their clinical counterpart for animal experiments still needs further investigation.

Practice guideline on “Management of hepatocellular carcinoma (HCC)” [15] and its update are the widely recognized guideline for the diagnosis and treatment for HCC [16–18]. To achieve the best outcomes requires the careful selection of candidates for each treatment option [6]. According to the guideline, different treatments should be applied for HCC in different stages; meanwhile, the same therapy may have variable outcomes for different staging tumors. It is reported that the necrosis rate of the HCC with the diameter of 2 cm, 2-3 cm and 3-5 cm by ethanol injection was 90-100%, 70% and 50%, respectively [19]. Lencioni et al. [20] reported that the efficacy of radiofrequency ablation (RFA) in tumors < 2 cm was similar to that of ethanol, but RFA was significantly better for tumors > 2 cm. In previous experiments, the treatment usually starts approximately 2 weeks after transplantation. Without taking individualization for experimental designs into consideration, the value of their outcomes may decrease to some extent. The purpose of this study was to investigate how to enroll the optimal animal model for the studies on HCC therapy.

### The biological progression of the VX2 liver tumor

The VX2 tumor is a highly malignant anaplastic squamous cell carcinoma. It is a fast-growing tumor with strong invasion, early hematogenous and lymphatic metastases and high lethality [21]. It was reported that the VX2 tumor followed the same metastatic pattern with HCC [22–26], therefore, the VX2 tumor was also used as a metastatic model of HCC [27]. This feature of fast progression has brought great challenge for selecting the optimal time for starting therapy. In this study, two commonly used inoculation methods including VX2 cell suspension and VX2 tissue fragment were used to establish animal models.

In our study, the tumor grew rapidly after implantation with a quick increase of the diameter and volume, leading to a shortened time span that was suitable for treatment. The animal model would have to be abandoned if the optimal intervention time was missed. The tumors derived from VX2 cell suspension and VX2 tissue fragment showed different growth behaviors. The tumors of the cell suspension group grew faster than those of the tissue fragment group. For animals inoculated using the tissue fragment method, the therapy starting point should be later than the one of animals inoculated using the cell suspension method.

The rate of tumor visualization on CT scans on day 14 after implantation was low. On day 7, the visualization rate on plain and contrast-enhanced CT scans was 12.5% and 53.13%, respectively. CT scans did not reliably detect the tumors at very early period after implantation. The blood supply of human HCC comes from the portal vein at its very early stage. As the tumor grows the blood supply becomes progressively arterialized [28]. The low visualization rate of CT scans may be due to the portal vein supply of the early-stage VX2 liver tumors or the improper enhanced phase used in CT enhanced scans. It is reported that the visualization rate of MRI scans was low at 1 week after implantation. the visualization rate of tumor on plain and contrast-enhanced MRI scans was 57.25% and 37.5%, respectively [29].

Metastases and ascites were not observed on day 7 in both groups. On day 14, one case in the cell suspension group showed intra-hepatic and pulmonary metastases. We considered that the multiple metastases may be due to dissemination though blood vessels which were broken during VX2 cell suspension injection. This is also mentioned in previous literature [30]. On day 21, metastases and ascites were observed in eight animals of the cell suspension group on CT scans. On day 28, most animals in the cell suspension group showed metastases and ascites. In the tissue fragment group, intra-hepatic metastases were only observed in one case on day 28. The average survival time of untreated animals was longer than 35 days. These results suggest that metastases and ascites might appear on approximately day 21 and 28, respectively in the cell suspension group and the tissue fragment group. Compared with the animals in the cell suspension group, the animals in the tissue fragment group indicated a longer survival time and a later occurrence of metastases and ascites.

Three reasons may contribute to the difference. Firstly, the tumors in the cell suspension group grew more fastly than those in the tissue fragment group, suggesting a more rapid disease progression; secondly, the way of tumor tissue implantation might influence its biological behavior; thirdly, the sample size of the tissue fragment group was comparably small.

### The optimal time to start therapy for the VX2 liver tumor

The BCLC system has been adopted as the international standard in HCC, which is recommended by both the American Association for the Study of Liver Diseases (AASLD) and the European Association for the Study of the Liver (EASL). This system uses variables related to tumor stage, physical status, liver functional status, and the extent of tumor spread. It has been suggested that it is best suited for treatment guidance, and particularly to select early-stage patients that could benefit from curative therapies [31,32].

The Child-Pugh score is used to assess the prognosis of chronic liver disease, mainly cirrhosis [33]. As a fast-growing xenograft model, rabbit VX2 liver tumor was lack of cirrhosis background. HCC animal model derived from chronic background, which is similar to the clinical situation of HCC patients, has not been reported. It was inappropriate for the Child-Pugh score assessing liver function on rabbit VX2 models. That is why the Child-Pugh score was not involved in this study. And we found repeated blood collection was harmful to the health of the rabbits in our preliminary experiment.

Patients in clinical trials should be staged by the BCLC system in order to provide meaningful comparisons between the outcomes reported and the prognosis of individual patients [6]. Most major trials [34–36] of HCC therapy have chosen the BCLC system, making it the de facto reference staging system.

**Figure 6:**
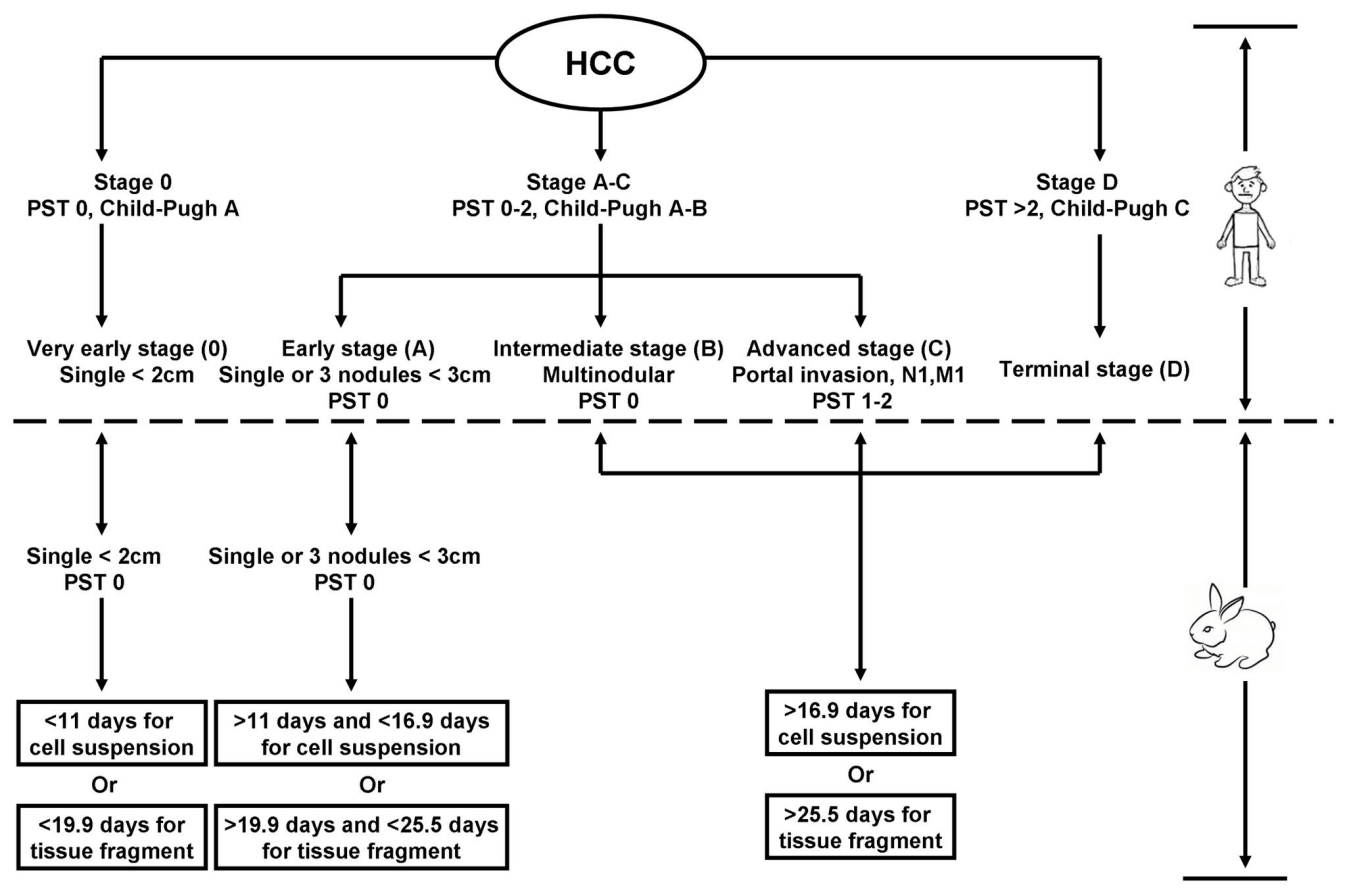
Comparisons of the VX2 staging with the BCLC staging. PST=Performance Status Test; N=lymph node; M=metastasis.

According to the BCLC system, HCC is divided into five stages including very early stage, early stage, intermediate stage, advanced stage and terminal stage. Therapeutic investigations usually focused on the HCC at very early, early and intermediate stages which may imply a relatively satisfactory efficacy [31,32]. Based on practice guideline of “management of hepatocellular carcinoma: update”, the BCLC system in combination with the biological progression of the VX2 liver tumor, we recommend a new staging proposal for the VX2 liver tumor (Figure 6). Very early stage means a single nodule (< 2 cm) without extra-hepatic metastases; early stage refers to a single nodule (2-3 cm) without extra-hepatic metastases; intermediate stage corresponds to a single nodule (>3 cm) without extra-hepatic metastases.

The time to tumor diameters of 2 cm and 3 cm was estimated by using the SPSS software. Good uniformity of tumor growth is the prerequisite for this method. Therefore, the cell suspension with the same cell number and the tissue fragments with the same volume should be applied.

On day 14, the tumor diameter of the cell suspension group and the tissue fragment group was 2.43 cm and 1.46 cm, respectively. Tumors with these two diameters correspond to different clinical stages. In previous studies, intervention usually started 2 weeks after implantation. Treatment start after two weeks of inoculation seems to be inappropriate, as individual VX2 tumors might be staged clinically unequally at this time point. Differences in clinical staging do not only concern tumor size but also presence of metastases and ascites. Therefore, treatment groups of animals might not match the HCC stages of their clinical counterparts.

The number of nodules, occurrence of extra-hepatic metastases and tumor diameter corresponding to clinical stages are the key factors when dividing animals into intervention groups. In addition, some potential requirement should also be taken into consideration according to study design. For instance, radiofrequency ablation on subcapsular liver tumor may lead to peritoneal dissemination [37], so the location and depth of tumor implantation should be carefully noted.

By applying a strict standard for enrolling animals to test groups, individual animals might be ruled out due to improper size of the primary tumor or the occurrence of metastases, thus leading to a reduced sample size. However, an optimal counterpart of human HCC patients would be selected for more reliable outcomes. Taking the attrition into consideration, designer should design a larger sample size in establishing models. According to this study, the recommended sample in establishing models should be 30% larger than that being used in intervention.

### Limitations

Our study has some limitations. First, as the rabbits could not survive repeated blood collection, liver function was not evaluated in this study. Second, the number of samples in the tissue fragment group was small due to other design of our series study. Third, the low sensitivity of CT scans in a relatively early period may lead to the low visualization rate of the intra-hepatic metastases. Fourth, although the blood supply of the VX2 liver tumor is similar to that of human HCC, the lack of cirrhosis background may limit the transition from the experimental results to clinical application.

To sum up, based on practice guideline of “management of hepatocellular carcinoma: update” and the BCLC system, we investigated how to select the optimal VX2 liver models tumor corresponding to different clinical stages. In order to obtain a reliable outcome, some key steps are required in preparing animals in HCC treatment studies using the VX2 rabbit model. Firstly, identify animal model stages according to the HCC clinical stages involved. Secondly, identify tumor transplantation method, and then the optimal intervention time. Thirdly, design proper sample size in establishing models according to the sample size being used in intervention. Each researcher should look for an individualized proposal according to study design.
